# An Exploration of the Inhibitory Mechanism of Rationally Screened Benzofuran-1,3,4-Oxadiazoles and-1,2,4-Triazoles as Inhibitors of NS5B RdRp Hepatitis C Virus through Pharmacoinformatic Approaches

**DOI:** 10.3390/biomedicines11113085

**Published:** 2023-11-17

**Authors:** Ali Irfan, Shah Faisal, Sajjad Ahmad, Muhammad Jawwad Saif, Ameer Fawad Zahoor, Samreen Gul Khan, Jamila Javid, Sami A. Al-Hussain, Muhammed Tilahun Muhammed, Magdi E. A. Zaki

**Affiliations:** 1 Department of Chemistry, Government College University Faisalabad, Faisalabad 38000, Pakistan; raialiirfan@gmail.com (A.I.); fawad.zahoor@gcuf.edu.pk (A.F.Z.);; 2 Department of Chemistry, Islamia College University Peshawar, Peshawar 25120, Pakistan; 3 Department of Health and Biological Sciences, Abasyn University Peshawar, Peshawar 25000, Pakistan; 4 Gilbert and Rose-Marie Chagoury School of Medicine, Lebanese American University, Beirut P.O. Box 36, Lebanon; 5 Department of Natural Sciences, Lebanese American University, Beirut P.O. Box 36, Lebanon; 6 Department of Applied Chemistry, Government College University Faisalabad, Faisalabad 38000, Pakistan; 7 Department of Chemistry, University of Sialkot, Sialkot 51040, Pakistan; 8 Department of Chemistry, College of Science, Imam Mohammad Ibn Saud Islamic University (IMSIU), Riyadh 13623, Saudi Arabia; 9 Department of Pharmaceutical Chemistry, Faculty of Pharmacy, Suleyman Demirel University, Isparta 32260, Turkey

**Keywords:** benzofuran derivatives, hepatitis C, RdRp NS5B inhibitors, molecular docking, MD simulations, MM-PBSA, SAR, ADMET studies, DFT studies, energy decomposition analysis

## Abstract

Benzofuran, 1,3,4-oxadiazole, and 1,2,4-triazole are privileged heterocyclic moieties that display the most promising and wide spectrum of biological activities against a wide variety of diseases. In the current study, benzofuran-1,3,4-oxadiazole **BF1**–**BF7** and benzofuran-1,2,4-triazole compounds **BF8**–**BF15** were tested against HCV NS5B RNA-dependent RNA polymerase (RdRp) utilizing structure-based screening via a computer-aided drug design (CADD) approach. A molecular docking approach was applied to evaluate the binding potential of benzofuran-appended 1,3,4-oxadiazole and 1,2,4-triazole **BF1**–**BF15** molecules. Benzofuran-1,3,4-oxadiazole scaffolds **BF1**–**BF7** showed lesser binding affinities (−12.63 to −14.04 Kcal/mol) than benzofuran-1,2,4-triazole scaffolds **BF8**–**BF15** (−14.11 to −16.09 Kcal/mol) against the HCV NS5B enzyme. Molecular docking studies revealed the excellent binding affinity scores exhibited by benzofuran-1,2,4-triazole structural motifs **BF-9** (−16.09 Kcal/mol), **BF-12** (−15.75 Kcal/mol), and **BF-13** (−15.82 Kcal/mol), respectively, which were comparatively better than benzofuran-based HCV NS5B inhibitors’ standard reference drug Nesbuvir (−15.42 Kcal/mol). A molecular dynamics simulation assay was also conducted to obtain valuable insights about the enzyme–compounds interaction profile and structural stability, which indicated the strong intermolecular energies of the **BF-9+NS5B** complex and the **BF-12+NS5B** complex as per the MM-PBSA method, while the **BF-12+NS5B** complex was the most stable system as per the MM-GBSA calculation. The drug-likeness and ADMET studies of all the benzofuran-1,2,4-triazole derivatives **BF8**–**BF15** revealed that these compounds possessed good medicinal chemistry profiles in agreement with all the evaluated parameters for being drugs. The molecular docking affinity scores, MM-PBSA/MM-GBSA and MD-simulation stability analysis, drug-likeness profiling, and ADMET study assessment indicated that *N*-4-fluorophenyl-*S*-linked benzofuran-1,2,4-triazole **BF-12** could be a future promising anti-HCV NS5B RdRp inhibitor therapeutic drug candidate that has a structural agreement with the Nesbuvir standard reference drug.

## 1. Introduction

The HCV (Hepatitis C virus) was first identified as a viral causative agent of Hepatitis infection in 1975 [1]. Hepatitis C virus (HCV), Zika, dengue, etc., are enveloped viruses that belong to the very famous human pathogen family Flaviviridae [2]. Hepatitis C virus infection has become a critical global health issue because 1.1% of the world’s population, or 71 million people, have Hepatitis C virus infection, which is a major cause of chronic liver diseases, as reported in a 2017 WHO report [3]. HCV, like other RNA viruses, has several non-structural proteins (NSPs). An essential and vital non-structural HCV protein in antiviral drug development is 5B (NS5B), a viral polymerase that carries out the transcription of HCV genomic RNA. Another critical non-structural protein is the NS3/4A protease, which plays a pivotal role in processing HCV viral polyproteins. Numerous inhibitors have been reported as effective therapeutic agents for managing HCV infection by targeting these important and crucial viral components of HCV [4,5]. These enzymes can be targeted by using various types of inhibitors, including nucleoside inhibitors (NIs) and non-nucleoside inhibitors (NNIs).

NIs are also termed direct-acting antiviral (DAA) inhibitors, which mainly target the active sites or the orthosteric sites of these vital viral targets. DAAs are competitive inhibitors, vying with the substrates of the active site for the enzyme catalytic site [6]. These inhibitors are frequently designed to target viral polymerases [7]. Unfortunately, the effectiveness of these inhibitors is compromised due to the higher rates of mutations occurring at the orthosteric sites within the viral proteins, leading to the emergence of viral drug resistance [8]. In contrast, NNIs, including allosteric inhibitors, have been employed extensively to address the issue of viral drug resistance [9]. Unlike NIs, which directly inhibit the active site of the NS5B polymerase, thereby terminating viral RNA synthesis, NNIs target allosteric sites within this enzyme, inhibiting its activity by alternate mechanisms. Nearly all NNIs function as allosteric inhibitors, obstructing the enzyme and impeding the required conformational changes vital for initiating RNA production, effectively inhibiting this enzyme activity [10].

These allosteric inhibitors provide a promising approach to tackling viral drug resistance and have the potential to be used safely in the treatment of a wide variety of viral infections. Pharmaceutical companies are actively involved in their production [11,12]. Similarly, there is extensive ongoing research aimed at discovering, identifying, and designing allosteric inhibitors targeting various viral proteins of the HCV virus [4,13,14]. One of the most targeted enzymes of HCV for allosteric drug discovery by researchers is the NS5B-viral polymerase. Researchers focus on this enzyme for allosteric drug discovery because it contains multiple allosteric sites that can be effectively targeted by various NNIs, thereby enabling efficient attenuation of HCV infection.

**Figure 1:**
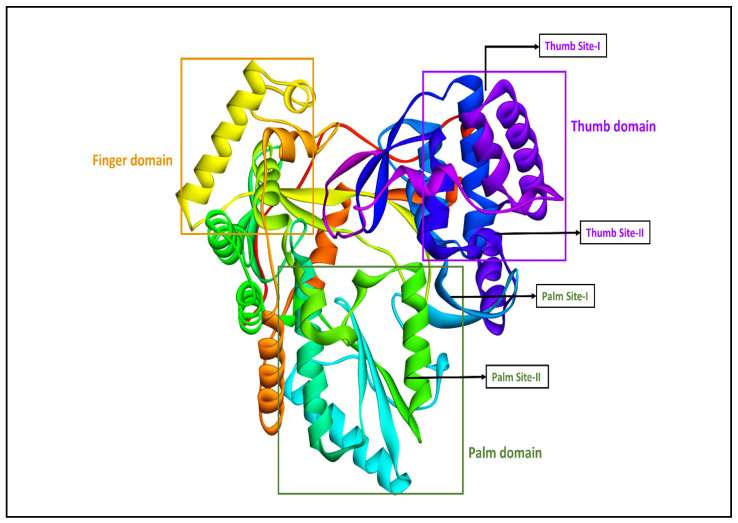
Structure of the HCV NS5B RNA-dependent RNA polymerase (PDB ID-4TLR) and the main allosteric regions for allosteric drug discovery.

The HCV virus’s NS5B RNA-dependent RNA polymerase has the same right-hand topology and contains the palm, finger, and thumb domains/regions like those of the other related viruses’ RdRp enzymes [15]. The NS5B enzyme of HCV has a thumb domain (Thumb Site-I and II), a palm domain (Palm Site-I and II), and a finger domain. These regions are highlighted and labeled one by one and can be viewed in Figure 1. The sub-regions in the thumb domain and palm domain, which are named Thumb Site-I and II (TS-I and II) and Palm Site-I and II (PS-I and II), respectively, are allosteric sites of the HCV NS5B enzyme. These four allosteric sites have been targeted by various types of compounds for allosteric inhibitors discovered against the HCV virus [4,13,14].

### 1.1. Heterocycles as HCV-NS5B Inhibitors

Heterocycles, especially nitrogen-containing compounds, have attracted considerable attention due to their wide spectrum and important biological uses in the fields of medicines, pharmacology, pharmaceutics, and pharmaceuticals. Heterocycles furans and benzofuran [16], pyrazoles [17], thiophenes [18], oxadiazoles [19], 1,2,4-Amino-triazines [20], thiadiazoles [21], imidazoles benzimidazoles, triazoles, pyridines, pyrimidines, benzoxazoles, benzothiazoles [22,23], etc., constitute the integral part of most of the clinical drugs and therapeutics to treat various diseases such as antiviral, anti-cancer, anti-bacterial, anti-allergic, anti-diabetic, analgesic, anti-histamine, anti-inflammatory, anti-tuberculosis, hemolytic, anti-convulsant, thrombolytic, anti-neurodegenerative, anti-leprosy, anti-tumor, antifungal, antipyretics, antihypertensive, dehydrogenase kinase inhibitor, herbicidal, etc. [16,17,18,19,20,21,22,23]. Heterocyclic structural hybrids of benzofurans, oxadiazoles, and triazoles display a wide spectrum of biological activities against various pathogenic and lethal diseases, especially the anti-HCV virus [24,25,26].

The four aforementioned allosteric sites in the HCV virus’s NS5B polymerase have been previously explored by different pharmaceutical companies and independent researchers in the quest for inhibitor discovery. Notably, William J. Watkins (2019) has reported several heterocyclic scaffolds featuring inhibitory compounds, such as benzofuran and indole scaffolds, that showed good inhibitory potencies against the NS5B of the HCV virus. The first reported allosteric site in HCV NS5B was TS-I, and it was explored for drug discovery by pharma companies such as Merck and Boehringer Ingelheim [27]. These companies utilized benzimidazole scaffolds for the inhibition of the HCV-NS5B (TS-I) allosteric site, which resulted in potent HCV inhibitors. Similarly, other pharma companies targeted the TS-II allosteric site of the HCV NS5B enzyme: ViroPharma/Wyeth, Agouron/Pfizer, GSK, and Gilead pursued designing allosteric drugs against the TS-II allosteric site [27]. They utilized benzamide, a thiophene-based scaffold, as well as other heterocycles like pyrrole and pyrazole-based compounds for drug design against HCV NS5B [27]. These efforts also yielded good allosteric inhibitors of the said target enzyme’s TS-II allosteric site. Similar intensive efforts were also made against the allosteric palm site regions for drug discovery against the NS5B of HCV [27].

**Figure 2:**
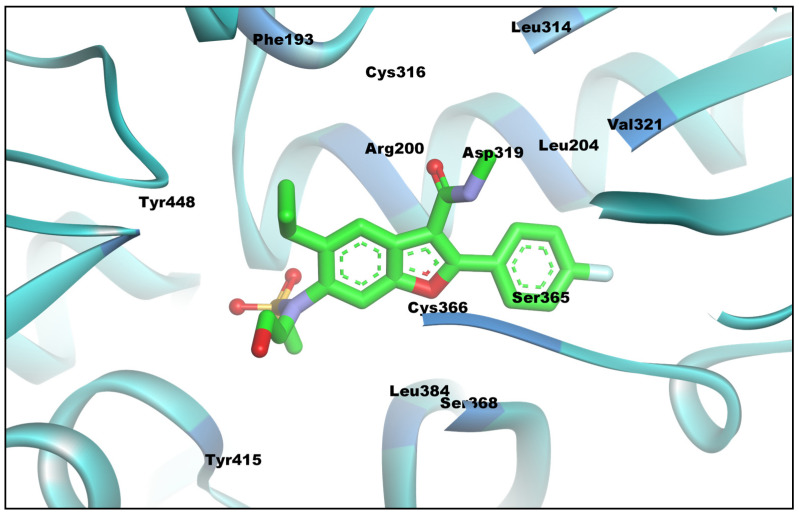
HCV NS5B Palm Site-II pocket allosteric residues with Nesbuvir inhibitor (PDB ID-4TLR).

Palm Site-I was explored by SmithKline Beecham, Anadys/Roche, and other companies, and they used different heterocycle-based compounds based on the benzothiadiazine pyrazolone and phosphadiazine-based scaffolds against the PS-I and further modifications and additions to these main scaffolds with other moieties like benzenesulfonamide, benzothiazine, naphthalene, and quinolone improved their efficacy against the HCV NS5B. Palm Site-II (PS-II) is also a hotspot for allosteric drug discovery. Similar approaches were used by pharma companies like ViroPharma/Wyeth, GSK, Merck, and Roche against the Palm Site-II, and they heavily utilized the benzofuran core [27] and the incorporation of substituents of diverse electronic effects on this benzofuran scaffold yielding potent allosteric drugs that also proved valuable in clinical interventions against the HCV viral infection. Some of the allosteric inhibitors developed against the HCV viral polymerase NS5B that we discussed above are provided in Figure 2 [28,29,30].

### 1.2. Rational Designed and SAR of Previously Reported Heterocycles

**Figure 3:**
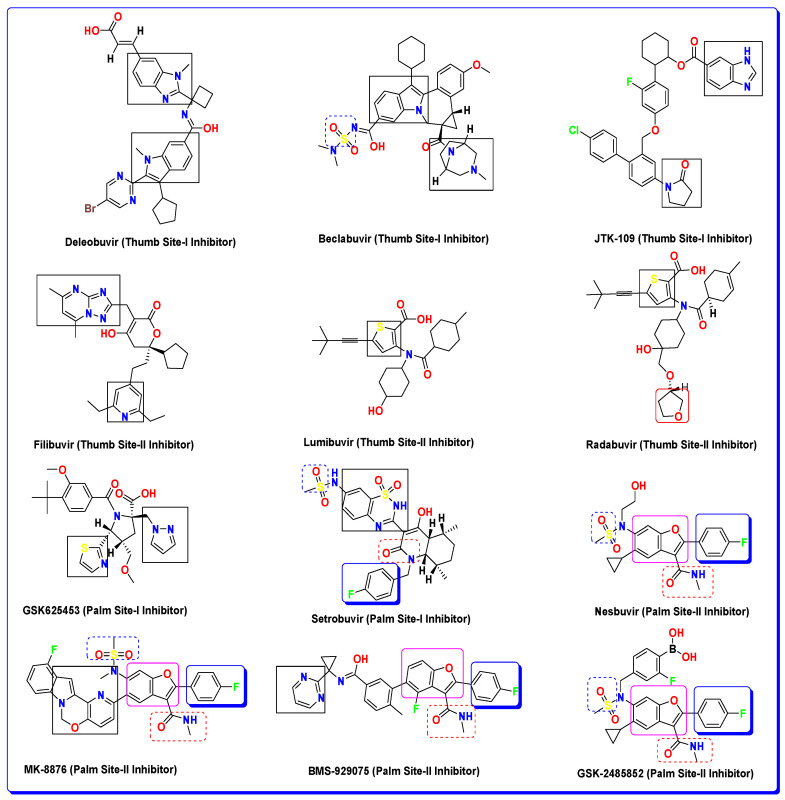
Allosteric inhibitors previously reported in the literature targeting different allosteric regions of the NS5B polymerase of HCV [26,27,28,29,30,31,32].

Previous SAR investigations of various inhibitors of Palm Site II have shown that this allosteric site has significant conformational flexibility and that it can accommodate diverse benzofuran-based compounds due to the plasticity/flexibility of this binding pocket [27]. The composition of this allosteric site, along with an inhibitor, Nesbuvir, is shown in Figure 3. Several amino acids that make up this site have been reported to be important for the binding of HCV NS5B inhibitors. These inhibitors interact with the allosteric site through a variety of molecular contacts, which ultimately inhibit the activity of this enzyme. Making molecular interactions with the SER365 and ARG200 allosteric residues has been reported to be very important for the inhibitory activity of Nesbuvir and its other benzofuran base derivatives, as these amino acid residues form key hydrogen bonding interactions with benzofuran-based compounds, e.g., Nesbuvir. Similarly, key interactions with CYS316 and CYS366 along with PHE193 of Nesbuvir further improve its inhibitory potency, and SAR studies have shown that engaging or making molecular interactions with these key Palm Site-II amino acids by an inhibitor is essential for inhibition and controlling the activity of the NS5B enzyme of HCV [31,32].

### 1.3. In Silico Anti-HCV NS5B Inhibitory Work Flow via CADD Approach

Other than these potent anti-HCV inhibitory activities of the benzofuran-based compounds, these benzofuran scaffolds have been reported to have a diverse set of activities against different types of enzyme targets implicated in various diseases. Inspired by the diverse bioactive profiles of benzofurans, oxadiazoles, and triazoles, we were interested in evaluating previously synthesized benzofuran derivatives (benzofuran–oxadiazoles and benzofuran–triazoles) [33,34] for their potential against the Hepatitis C virus via an in silico structure-based computer-aided drug design (CADD) approach to evaluate the affinities and binding potential with the Palm Site-II (PS-II) of the HCV RdRp enzyme.

**Figure 4:**
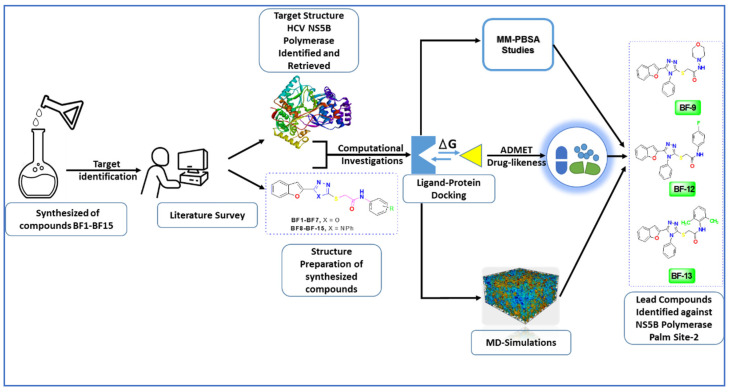
Workflow of anti-HCV NS5B inhibitors drug discovery via CADD approach.

The assessment of the therapeutic efficacy of synthesized benzofuran-1,3,4-oxadiazole and 1,2,4-triazole scaffolds was carried out by applying the CADD approach, as depicted in Figure 4.

## 2. Materials and Methods

### 2.1. Chemistry of Benzofuran-1,3,4-Oxadiazoles BF1-7 and-1,2,4-Triazoles BF8-15

All the screened benzofuran–oxadiazole and triazole structural motifs BF1–15 were synthesized and published [33,34], and their structural formulae are provided in Supplementary Table S1.

### 2.2. Molecular Docking of Benzofuran-1,3,4-Oxadiazoles BF1-7 and-1,2,4-Triazoles BF8-15

The molecular docking computational research was carried out using the PDB structure of the target enzyme HCV NS5B, with PBD code 4TLR [35] downloaded from the RCSB website. The Molecular Operating Environment (MOE) was used to carry out the molecular docking analyses (Version 2009.10). The target enzyme’s protein structure was prepared for docking investigations using the Biovia DS program [36]. ChemDraw Professional was used to prepare the ligands’ structures, and then these ligands’ structures in (.mol) format were imported into MOE, where the partial charges were incorporated into it, and the compounds’ energies were minimized using the MMFF94x-ff. The protein structures were loaded and 3D protonated in MOE, and the site-finder function was then used to determine the allosteric pocket of the HCV NS5B enzyme [37]. The DOCK module of MOE software and the triangle matcher approach, along with the London-dG scoring algorithms, were selected to estimate the binding affinity of these compounds against the HCV NS5B enzyme. Furthermore, the ligand–protein complexes’ interactions were analyzed and visualized using the BIOVIA DS software.

### 2.3. ADMET and Drug-Likeness Studies of Benzofuran-1,3,4-Oxadiazoles BF1-7 and-1,2,4-Triazoles BF8-15

The drug-likeness, ADMET, and medicinal chemistry profiles of the compounds were predicted using the ADMETlab2.0 online server [38].

### 2.4. MD Simulation of the Most Bioactive BF-9, BF-12 and BF-13 Derivatives

The simulation of docked complexes was accomplished using AMBER20 [39]. The initial parameterization of docked systems was processed using the Antechamber program. The force fields used to describe the HCV NS5B enzyme and compounds were FF14SB and GAFF, respectively [40,41]. All the simulated complexes were submerged into a TIP3 solvation box (padding distance of 12 Å). The systems were neutralized by adding counterions. The Particle–Ewald summation method [42] was used to define long-range electrostatic interaction. The simulation was carried out in four phases: first, energy minimization via two algorithms in a sequential manner (steepest descent and conjugate gradient), heating to 310 K, followed by equilibration and a production run for 100 ns. In the production run, constraints on bounded hydrogen atoms were accomplished using the SHAKE algorithm, while the temperature was maintained by Langevin [43,44]. The simulation trajectories were generated by employing NVE and NPT ensembles and setting the collision frequency to 2. The simulation trajectories were investigated using the CPPTRAJ module [45], and the plots were generated and analyzed in XMGRACE 5.1 [46].

### 2.5. MM-PBSA Binding Free Energy Calculations of the Most Bioactive BF-9, BF-12, and BF-13 Derivatives

The estimation of binding free energy docked compounds with HCV NS5B was carried out using the Molecular Mechanics Poisson–Boltzmann Surface Area (MM-PBSA) and Molecular Mechanics Generalized Born Surface Area methods [47]. The initial files and processing were carried out using the AMBER20 MMPBSA.py method [48]. The energy calculation was carried out throughout the length of the simulation time, considering 1000 simulation frames. The binding free energy was estimated using Equation (1).

ΔG _net binding energy_ = G_protein-ligand complex_ − (G_protein_ + G_ligand_) (1)

### 2.6. DFT Studies of the Most Bioactive BF-9, BF-12, and BF-13 Derivatives

The DFT study of **BF-9**, **BF-12**, and **BF-13** was performed using the Gaussian program, as reported earlier [49,50]. The total energy, the highest occupied molecular orbital (HOMO) energy, and the lowest unoccupied molecular orbital (LUMO) energy were obtained from the Gaussian program, and the related energies were also calculated. Thereafter, the calculated energies were interpreted accordingly.

## 3. Results and Discussion

### 3.1. Chemistry

The chemical structures of seven benzofuran-1,3,4-oxadiazoles **BF1**–**BF7** and eight benzofuran-1,2,4-triazoles **BF8**–**BF15** have been provided in Supplementary Table S1.

### 3.2. Computational Biological Screening of Benzofuran-1,3,4-Oxadiazoles BF1–BF7 and-1,2,4-Triazoles BF8–BF15 Using CADD Approach

The in silico molecular docking investigation of these fifteen benzofuran-linked oxadiazole and triazole compounds was performed against the HCV RdRp important enzyme to evaluate their binding affinities using the MOE software. The studies revealed that the evaluated benzofuran compounds (benzofuran–oxadiazole hybrids) and the benzofuran–triazole hybrids were able to show good affinities with the Palm Site-II of the HCV NS5B. The synthesized benzofurans (BF1–BF15) showed significantly improved and comparable binding affinities to the PS-II allosteric site of HCV NS5B compared to the standard inhibitor Nesbuvir, as determined by molecular docking studies, as shown in Supplementary Table S1.

**Figure 5:**
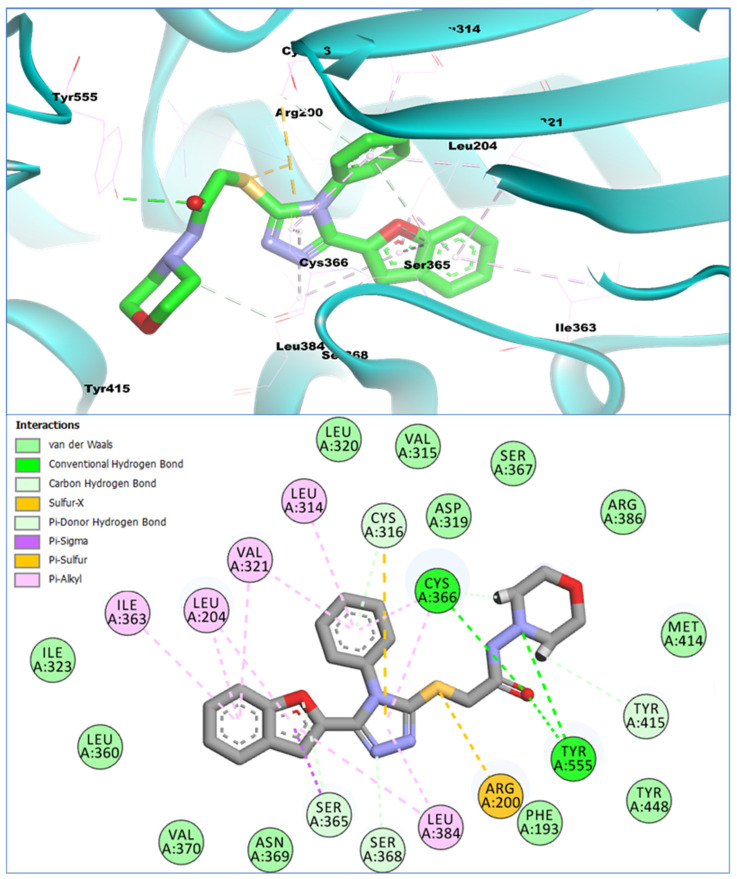
The binding conformation of **BF-9** in 3D (**upper panel**) and 2D (**lower panel**) with the HCV NS5B PS-II allosteric site.

**Figure 6:**
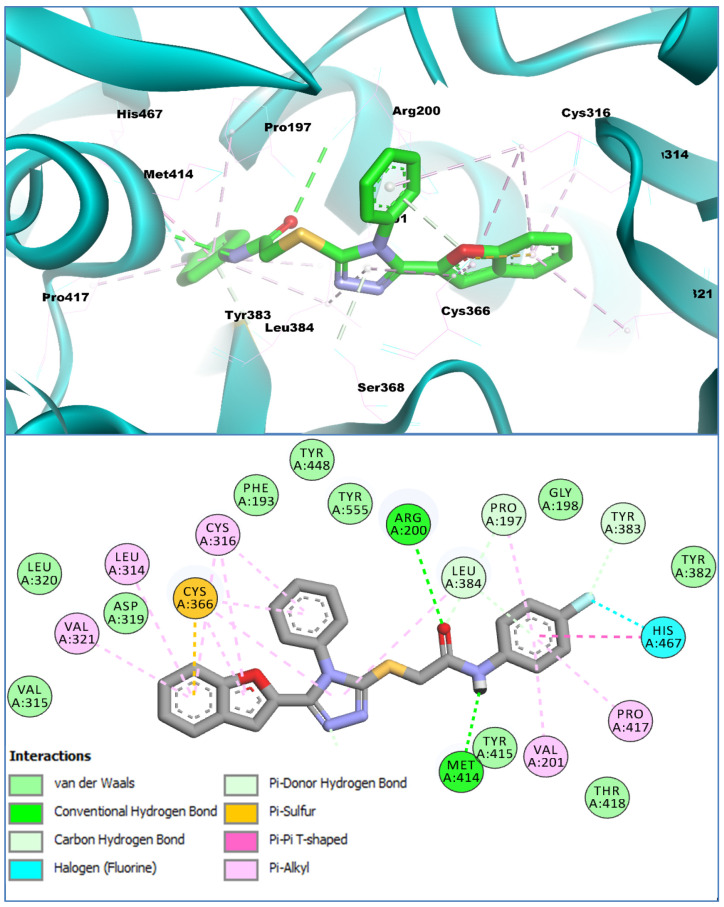
The binding conformation of **BF12** in 3D (**upper panel**) and 2D (**lower panel**) with the HCV NS5B PS-II allosteric site.

The binding affinity analysis of the standard inhibitor of HCV, Nesbuvir, with the NS5B (PS-II) site, revealed that it bound to this site with a binding affinity of −15.42 Kcal/mol. Compared to the Nesbuvir compound, only 3 out of the 15 reported benzofurans showed comparable binding affinities with the target enzyme. The **BF-9** benzofuran–oxadiazole hybrid compound containing morpholinyl structural moiety was able to show a significantly higher binding affinity with the PS-II allosteric site of the HCV NS5B. It showed a binding affinity of −16.09 Kcal/mol with the target enzyme, while the conformational analysis of this compound with the PS-II site revealed that it occupied this allosteric pocket with a stable conformation and had significant molecular interactions with the allosteric pocket residues. It can be seen in Figure 5 that **BF-9** interacts with the allosteric pocket residues via conventional and carbon–hydrogen-type hydrogen bonds. CYS316 and SER368 can be seen in Figure 6, which shows the C-H type hydrogen bond with the phenyl ring and the triazole ring’s nitrogen atom, respectively.

The furan core and the morpholine moiety of the **BF-9** also contributed to a hydrogen bond by engaging the SER365 and the TYR415 allosteric pocket residues. The acetamide scaffold’s oxygen atom, as well as the nitrogen atom of the morpholine moiety present in this compound, were able to engage multiple residues via conventional hydrogen bonding. The sulfur-containing thio group of **BF-9** also interacted with the allosteric pocket via sulfur-X and Pi-Sulfur interactions. Other non-covalent interactions, i.e., pi-sigma, pi-alkyl, van der Waals, etc., that further stabilize a compound inside a protein pocket were also observed in the **BF-9** and HCV NS5B enzyme complexes. These higher interactions and affinity of **BF-9** suggest its stability within the PS-II allosteric pocket of HCV NS5B. Its conformational 2- and 3-dimensional poses are provided in Figure 5.

Similarly, **BF-12** (having a 4-fluorophenyl moiety attached to the acetamide scaffold) was also able to show higher affinity with the HCV NS5B PS-II allosteric pocket than Nesbuvir. **The BF-12** structural motif showed a binding affinity of −15.75 Kcal/mol with the HCV NS5B PS-II allosteric pocket; its binding conformation, which is provided in Figure 5, shows that **BF-12** binds with the PS-II pocket of HCV NS5B with a similar binding pose to that of the **BF-9** structural motif. The interaction analysis of **BF-12** revealed that it made conventional and carbon–hydrogen-type H-bonds with the CYS384, SER368, PRO197, and TYR383 of the PS-II allosteric pocket. Similarly, the acetamide scaffold also showed stronger conventional-type hydrogen bonding by interacting with the ARG200 and MET414 of the PS-II of the HCV NS5B enzyme. Other stabilizing interactions of different types that were present in the **BF9-NS5B** complex were also present in the **BF12-NS5B** ligand enzyme complex, while the fluorophenyl moiety of **BF-12** along with the benzofuran core of **BF-12** contributed and showed significantly higher molecular interactions with the PS-II allosteric pocket, indicating higher affinity and stability inside the PS-II of the HCV NS5B enzyme. Its conformational poses are presented in Figure 6.

**Figure 7:**
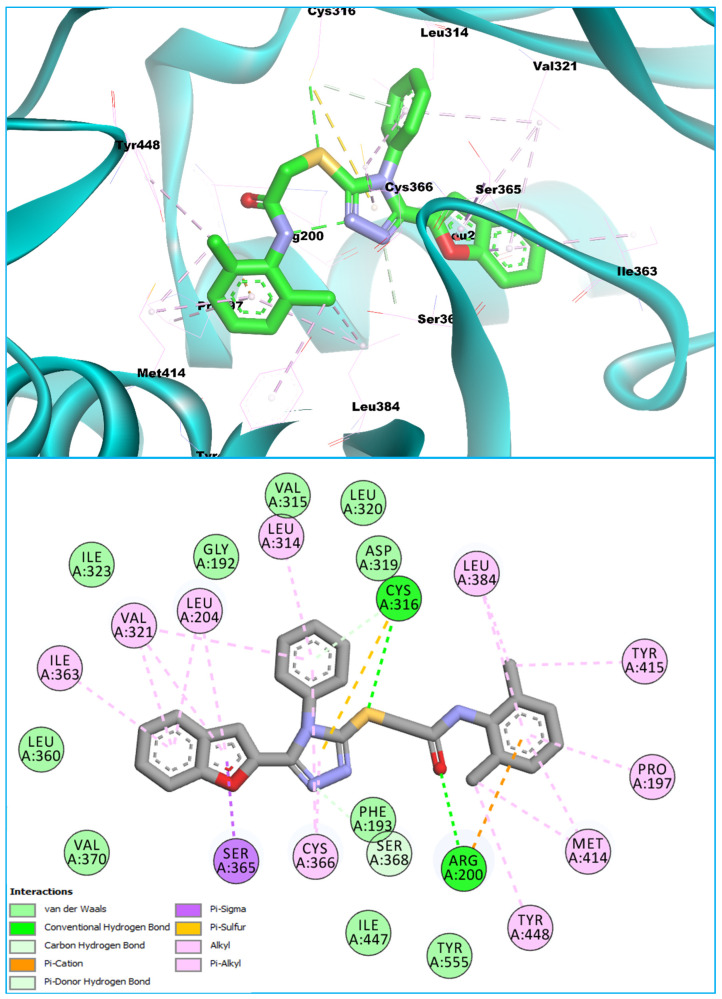
The binding conformation of **BF13** in 3D (**upper panel**) and 2D (**lower panel**) with the HCV NS5B PS-II allosteric site.

Another compound, **BF-13** (belonging to the benzofuran–triazole-based class of compounds), showed higher affinities towards the HCV NS5B PS-II allosteric site. The scaffold **BF-13** showed an affinity of −15.82 Kcal/mol with the PS-II allosteric site of the HCV NS5B enzyme and showed robust molecular interactions with the target enzyme allosteric site’s pocket residues. Compound **BF-13** showed a similar binding conformation as that observed in the BF9-NS5B ligand–protein complex. The structural hybrid **BF-13** showed diverse types of molecular interactions with the PS-II allosteric site by engaging the allosteric pocket residues via conventional as well as carbon–hydrogen-type hydrogen bonding interactions. The acetamide, as well as the thio group, also showed good interactions with the allosteric site residues. The benzofuran core, the triazole scaffold, and the dimethyl phenyl moiety also contributed to various types of diverse molecular interactions with the target enzyme’s (PS-II) allosteric site. Figure 7 shows the 2- and 3-dimensional conformations of BF-13 with HCV NS5B.

Furthermore, compounds having different substituents studied against the HCV NS5B PS-II allosteric domain showed considerable differences in affinity towards this target enzyme. Notably, within these benzofuran compounds featuring nitrogen-based heterocycles such as oxadiazole and triazole, there was a consistent pattern where compounds containing a triazole moiety (benzofuran–triazoles) exhibited a considerably higher affinity for the target enzyme compared to those with benzofuran–oxadiazoles.

The literature reveals that there are significant differences between an enzyme active site, also known as the orthosteric site, and the allosteric sites present in it. It is reported in the literature that the active/orthosteric site receptor amino acid residues tend to be more polar, while the amino acid residues present in the allosteric site are hydrophobic in nature [51,52,53,54]. These differences in the amino acid residues in the active site and allosteric site affect the ligands/inhibitors/modulators that bind to these sites in an enzyme. Further investigations showed that the compounds in the allosteric inhibitors/modulators that target allosteric sites are more aromatic and more rigid as compared to those inhibitors that target the active sites [55,56]. From these observations, we deduced that benzofuran compounds having the triazole moiety substituted with an extra-phenyl (which increases their hydrophobicity) increase their affinity with the target enzyme as opposed to benzofurans with the corresponding oxadiazole moiety. The compounds that had the highest binding affinities to the HCV NS5B PS-II allosteric site are listed in Table 1.

Moreover, previous literature investigations into the inhibitor discovery targeting Plam Site-II of the HCV NS5B polymerase have shown some of the important pharmacophoric features of Plam Site-II inhibitors. The reported inhibitors of this specific site mainly consist of the benzofuran core scaffold (already discussed in the introduction section). SAR studies on these benzofuran-based Palm Site-II inhibitors have shown the important receptor residues of this site. Nesbuvir (HCV-796) has been shown to form important interactions with certain residues of this site and is considered important to be engaged by inhibitors targeting this site. Nesbuvir and its derivatives [31], along with BMS-929075, another benzofuran Palm Site-II inhibitor [31,32], and other developed inhibitors bearing a fused benzofuran scaffold [57], have been reported to have a similar interaction pattern with some specific allosteric residues of the Palm Site-II. These inhibitors have been reported to engage SER365 and ARG200 with key hydrogen bonding interactions; similarly, several changes in the benzofuran side groups that were able to engage CYS316, CYS366, and PHE193 pocket residues have been reported to increase the potencies of these inhibitors against the NS5B polymerase [31]. In comparison to these reported benzofuran-based inhibitors, the compounds investigated here also showed diverse types of strong interactions with the targeted allosteric site of NS5B polymerase. The benzofuran core of **BF-9** can be seen in Figure 5, engaging different allosteric pocket residues. Similarly, the triazole moiety and the substituted phenyl on this ring can be seen making diverse interactions of both hydrogen bonds as well as other hydrophobic interactions. The sulfur atom, along with the acetamide moiety, can also be seen making significant and important interactions with the receptor residues. These similar binding interaction patterns, along with the comparable binding affinities of these compounds with previously reported inhibitors against the HCV NS5B polymerase Palm Site-II, suggest that these compounds have a promising potential for the development of new drugs to treat HCV infection. Further studies are needed to confirm these findings and evaluate the efficacy and safety of these compounds in animal models and humans.

### 3.3. Structure–Activity Relationship (SAR) of the Most Bioactive Benzofurans BF-9, BF-12, and BF-13

We rationally evaluated a set of *S*-alkylated *N*-phenyl-based benzofuran–triazoles and oxadiazoles by considering the incorporation of various moieties, functionalities, and features found in well-established reference antiviral drugs, as illustrated in Figure 3. The prevalent antiviral constituents in all the drugs featured in Figure 3, particularly the Nesbuvir reference drug, encompass oxygen-based heterocycles, nitrogenous heterocycles, substituted phenyls, sulfur, and amide groups.

These assessed derivatives possess a core structure comprising a benzofuran, serving as an oxygen-based heterocycle, in addition to oxadiazole and triazole functionalities as nitrogenous heterocyclic components. They also incorporate sulfur, amide groups, and substituted *N*-phenyl units within their framework. These structural features validate their potential as chemotherapeutic agents against various viral targets.

**Figure 8:**
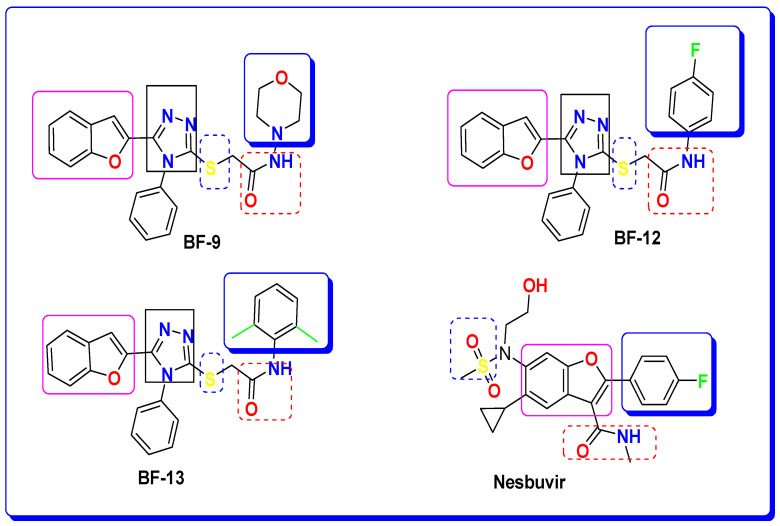
SAR of benzofuran-1,2,4-triazole **BF-9**, **BF-12,** and **BF-14** hybrid structures.

In our study, the results obtained through molecular docking techniques revealed that the presence of the polynitrogen triazole ring, sulfur and amide moieties, and the *N*-phenyl ring in the structures of the most potent antiviral benzofurans (BF-9, BF-12, and BF-13) exhibited significantly improved and comparable binding affinities compared to the Nesbuvir standard drug. Notably, this improvement was particularly evident in comparison to Nesbuvir, which lacks the triazole moiety, as depicted in Figure 8.

### 3.4. ADMET and Drug-Likeness Investigations of the Most Bioactive Benzofurans BF-9, BF-12, and BF-13

These compounds were further evaluated for their pharmacokinetics and drug-likeness properties and showed favorable physicochemical properties and LogS, LogD, and LogP scores. These compounds had optimal molecular weights and TPSA scores, along with other physicochemical properties like the number of hydrogen bond acceptors and donors, as well as the number of rings (nHA, nHB, and nRing) present in them that are necessary for a bioactive compound. These compounds showed low to medium permeability in the MDCK cells and showed optimal human intestinal absorptions (HIA), as predicted by ADMETlab 2.0. All of the compounds showed good medicinal chemistry profiles and completely complied with Lipinski’s Rule and the Golden Triangle medicinal chemistry rules.

The toxicity studies showed that only the **BF9** compound is not AMES toxic, and the other two are AMES toxic. Moreover, these compounds have low rat oral toxicity along with lower hepatotoxicity profiles. The acute toxicity rule and aquatic toxicity rule also showed no alerts for these compounds. The predictive metabolism studies revealed that some of these compounds are non-inhibitors of the CYP1A2, CYP2C9, and CYP2D6 and are substrates of the CYP2C19 and CYP3A4 metabolic transformation enzymes. The excretion prediction profiles of these compounds showed they show moderate to low clearance values (5–15 mL/min/kg) from the host excretory system. ADMET and drug-likeness profile information for the top three compounds is provided in Table 2.

### 3.5. MD Simulations of the Most Bioactive Benzofurans BF-9, BF-12, and BF-13

**Figure 9:**
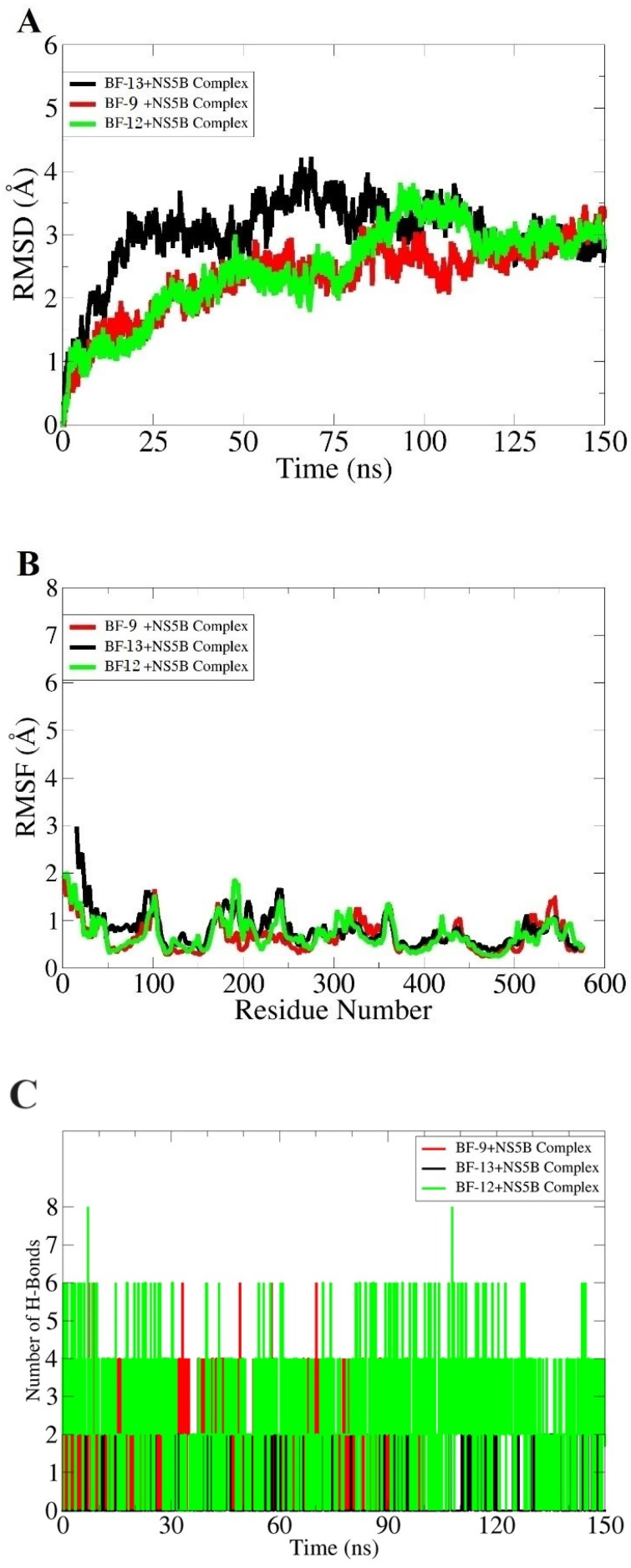
Analysis based on molecular dynamics simulation. (**A**) RMSD, (**B**) RMSF, (**C**) intermolecular hydrogen bonds analysis.

The molecular dynamics simulation assay was conducted to obtain valuable insights into the enzyme–compound interaction profile and structural stability along the simulation time. The impact of compounds on the enzyme’s conformational stability, root mean square deviation (RMSD), was computed based on backbone carbon alpha atoms (Figure 9A). The **BF-13+NS5B** complex showed more deviations compared to the other two (**BF-12+NS5B** complex and **BF-9+NS5B** complex) until 125 ns. The RMSD of this system touches almost 4 angstroms at 75 ns. Initially, the system revealed an increasing RMSD pattern in the first 30 ns phase, then gained some stability and was subjected to another round of high deviation. After 125 ns, the RMSD can be seen to have consistent structural stability. Similarly, the **BF-12+NS5B** complex and the **BF-9+NS5B** complex experienced structure fluctuations until 125 ns but then equilibrated till the end. The residue level fluctuations of HCV NS5B in the docked systems were revealed through root mean square fluctuation (RMSF), as displayed in Figure 9B. The C-terminal residues were more flexible compared to the rest of the enzyme structure. The majority of the enzyme residues are within a stable range, and the presence of compounds does not influence enzyme conformation stability. In the case of BF-9+NS5B, the RMSF values of the active site residues were Cys366 (1.12 Å) and Tyr555 (1.14 Å). Similarly, BF-12+NS5B reported the following active site residues as the most stable, with RMSF values of Arg200 (0.86 Å) and Met414 (0.67 Å). The RMSF values of residues involved in BF-13+NS5B hydrogen bond interactions were Arg200 (0.97 Å) and Cys316 (1.1 Å). Throughout the length of the simulation time, the intermolecular interaction network of the complexes was found to be uniform, with no major deviations. This can be inferred from the constant RMSD plots. Further, the number of hydrogen bonds formed between the compounds and the enzyme was estimated using the VMD H-Bonds plugin. It can be seen in Figure 9C that all complexes reported the formation of several intermolecular hydrogen bonds along the length of simulation time, supporting intermolecular conformational stability. The BF-12+NS5B complex, in particular, was noticed to show a high number of intermolecular hydrogen bonds in the simulation time.

### 3.6. MM-PBSA Investigations of the Most Bioactive Benzofurans BF-9, BF-12, and BF-13

The binding free energy calculation was carried out to obtain insights into the compound’s binding potential with the receptor, both in bounded and unbounded states. In the calculations as tabulated in Table 3, it can be noticed that all the compounds showed robust atomic-level interaction energies with NS5B. The major dominance was seen in gas-phase energy, which can be split into van der Waals energy and electrostatic energy. The van der Waals component was observed to dominate the net energy contribution. The electrostatic energy also played a significant role in the docked compounds’ stabilization with the enzyme. This illustrates that the majority of the chemical regions of the compounds are bridged to the enzyme-active residues through hydrophobic contacts. The hydrophilic contacts supported the intermolecular interactions and stabilized the binding mode of the compounds to the enzyme-active pocket. According to MM-GBSA analysis, the net binding free energy of **BF-9+NS5B** complex, **BF-12+NS5B** complex, and **BF-13+NS5B** complex is −77.33 Kcal/mol, −78.16 Kcal/mol, and −71.31 Kcal/mol, respectively (Table 3). This shows that the **BF-12+NS5B** complex is the most stable system as per the MM-GBSA calculation. The mean Van der Waals and electrostatic energies of this system are −69.25 Kcal/mol and −28.09 Kcal/mol, respectively. The **BF-9+NS5B** complex and the **BF-12+NS5B** complex showed strong intermolecular energies as per the MM-PBSA method, with a binding energy of less than −75.40 Kcal/mol.

### 3.7. Energy Decomposition Analysis

Further, the MM-GBSA net energy was decomposed into residue-wise energy in order to highlight the residues that contributed significantly to the compounds binding with the enzyme. The residues that have a binding energy score of <−1 Kcal/mol were tagged as hotspot residues due to their good stability in the presence of ligands. The free energy decomposition analysis results are provided in Table 4. According to the data, Arg200, Cys366, and Met414 were the most contributing residues that strongly bridged the enzyme to the compounds.

### 3.8. DFT Studies of the Most Bioactive Benzofurans BF-9, BF-12, and BF-13

The HOMO and LUMO energy values of **BF-9, BF-12**, and **BF-13** that were obtained from the DFT calculation were utilized to calculate the other related parameters. The parameters computed and the formulas used are provided in Table 5 [58,59].

The DFT study exhibited that a significant difference in the electrochemical properties of the investigated compounds was not observed. Since these compounds are derivatives of a core structure without major variation, the similarity of their electrochemical properties was as expected. Together with this, the small difference in the parameters calculated was analyzed in a way that would make sense to the findings. First, the relative electron exchange capability of the three derivatives was evaluated by the HOMO and LUMO energies. The HOMO energy value of **BF-13** was the highest in the DFT study (Table 4). Since HOMO is representative of the electron-donating capability of a compound, **BF-13** is expected to have the highest electron-donating tendency [63]. On the other hand, the LUMO energy value of **BF-12** was found to be the highest (Table 4). As LUMO is representative of electron-accepting capability, **BF-12** is anticipated to give electrons easily relative to the other two derivatives [64]. The relative stability of compounds can be evaluated by using their HOMO-LUMO energy gaps. A higher energy gap implies higher chemical stability for compounds [65].

**Figure 10:**
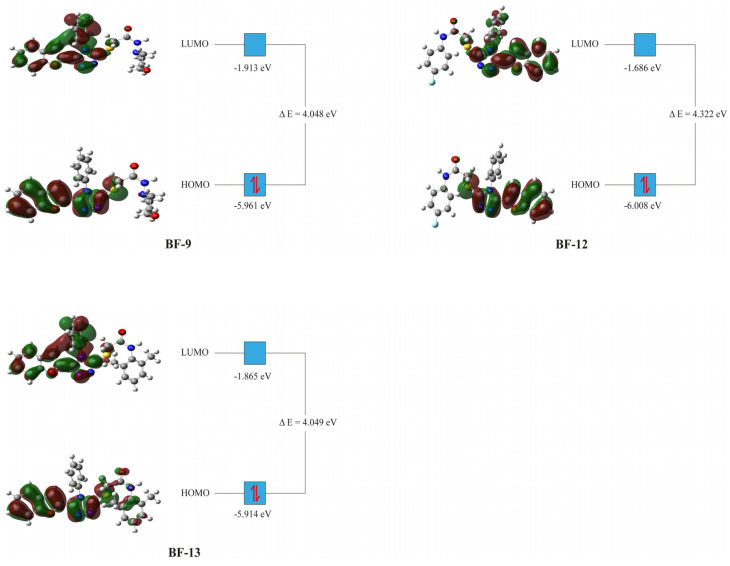
Molecular orbital orientations of compounds **BF-9**, **BF-12**, and **BF-13** at LanL2DZ/B3LYP DFT levels in the ground state.

The DFT study results showed that **BF-12** had the highest energy gap (Table 4). Therefore, compound **BF-12** is expected to possess the highest chemical stability. A lower energy gap provides a higher ease of charge transfer and, thus, a higher chemical reactivity for a compound. Therefore, **BF-9** is expected to have the highest reactivity. Together with this, the compounds produced similar energy gaps that would imply similar stability and reactivity for them (Table 5, Figure 10). On the other hand, the resistance of atoms to electron transfer is represented by global hardness. In this study, **BF-12** had the highest global hardness value (Table 4). From these results, it is possible to infer that compound **BF-12** might have the highest chemical stability and the least reactivity [66].

The HOMO-LUMO orbital orientations for compounds **BF-9**, **BF-12**, and **BF-13** were similar to each other (Figure 10). The HOMO orbitals of compounds **BF-9** and **BF-12** were concentrated on benzofuran, triazole, and the functional group bridge. In addition to the HOMO orbital concentrations of **BF-9** and **BF-12**, compound **BF-13** had similar orbitals on the phenyl group substituted for the amine group, but the orbital density was sparse here (Figure 10). The LUMO orbitals of **BF-9**, **BF-12**, and **BF-13** were mainly concentrated on the vicinities of benzofuran, triazole, and the phenyl substituted to the triazole ring. Furthermore, sparse LUMO orbitals were observed around the sulfur atom. There was a similarity between the DFT study results and the interactions detected via molecular docking. In the DFT study, potential electron exchange vicinities were observed around the benzofuran, triazole, the phenyl substituted to it, and the functional group bridge. Similarly, various interactions were observed between the compounds and the enzyme in these vicinities. The difference in the concentration of electron exchange potential areas and interaction points was observed on the substituted phenyl ring next to the bridge functional group. Though there was no orbital concentration on it, various interactions were observed between the compounds and the enzyme (Figure 5, Figure 6 and Figure 7 and Figure 10).

## 4. Conclusions

In the present study, benzofuran-1,3,4-oxadiazole **BF1**–**BF7** and benzofuran-1,2,4-triazole compounds **BF8**–**BF15** were virtually screened against HCV NS5B RdRp enzymes via the CADD approach. In silico structure-based computer-aided drug design methodology was applied to evaluate the affinities and binding potential of benzofuran-1,3,4-oxadiazole and 1,2,4-triazole **BF1**–**BF15** hybrid structures. The benzofuran-1,2,4-triazoles **BF8**–**BF15** showed excellent and remarkably high affinities and binding scores (−14.11 to −16.09 Kcal/mol) against the HCV NS5B enzyme in comparison to their sister benzofuran-1,3,4-oxadiazole molecules (−12.63 to −14.04 Kcal/mol) as well as the Nesbuvir standard reference drug due to the presence of a triazole ring. The highest binding affinity scores were displayed by the benzofuran-1,2,4-triazole structural motifs **BF-9** (−16.09 Kcal/mol), **BF-12** (−15.75 Kcal/mol), and **BF-13** (−15.82 Kcal/mol) amongst all fifteen compounds **BF1**–**BF-15,** as well as by Nesbuvir (−15.42 Kcal/mol). The molecular dynamics simulations were conducted to obtain valuable insights about the enzyme–compound interaction profile and structural stability, which indicated that the **BF-9+NS5B** complex and the **BF-12+NS5B** complex showed strong intermolecular energies as per the MM-PBSA/MM-GBSA method with a binding energy of less than −75.40 Kcal/mol, while the **BF-12+NS5B** complex is the most stable system as per the MM-PBSA/MM-GBSA calculations. The results of the MM-PBSA/MM-GBSA calculations reveal a significant enhancement in the free binding affinity energies of the studied complexes when compared to the docking energies. This implies a strong attraction between these compounds and the overall stability of the complexes. In particular, both the **BF-9+NS5B** complex and the **BF-12+NS5B** complex demonstrate significant intermolecular interactions, with a binding energy below −75.40 Kcal/mol. Among them, the **BF-12+NS5B** complex emerges as the most robust system based on the calculations using MM-PBSA/MM-GBSA. These findings indicate a high affinity of these compounds for the Palm Site-II binding pocket of the NS5B polymerase of HCV. The drug-likeness and ADMET studies of all the benzofuran-1,2,4-triazole derivatives **BF8–BF15** revealed that these compounds demonstrated promising medicinal chemistry profiles in agreement with all evaluated parameters for being drugs. The molecular docking binding affinity score, MM-PBSA/MM-GBSA, MD-simulation stability analysis, drug-likeness profiling, and ADMET assessment results indicated that *N*-4-fluorophenyl-*S*-linked benzofuran-1,2,4-triazole **BF-12** could be a promising future inhibitor of HCV NS5B RdRp enzyme, which has therapeutic potential to be a leading drug candidate.

## Figures and Tables

**Table 1:** Binding affinities of the lead compounds and the Nesbuvir (standard) in the molecular docking studies, along with the molecular interactions observed with the HCV NS5B Palm Site-II allosteric residues.

Compounds	Binding Affinities with NS5B PS-II (Kcal/mol)	Hydrogen Bonds with PS-II Residues	Hydrophobic Interactions with PS-II Residues	Other Interactions, e.g., Sulfur-X, Pi-Sulfur, and Halogen Interactions with PS-II Residues
**BF-9**	−16.09	CYS316, SER365, CYS366,SER368, TYR415	LEU204, LEU 314,VAL321, ILE363,SER365, CYS366, LEU384	CYS316, CYS366, ARG200, TYR555
**BF-12**	−15.75	CYS366, SER368, LEU384,PRO197, LEU384, TYR383, ARG200	VAL201,LEU314, VAL321, CYS316, PRO417, HIS467, CYS366, LEU384	HIS467, MET414,
**BF-13**	−15.82	CYS316, ARG200, SER368,	LEU204, LEU 314, VAL321, ILE363, SER365, CYS366, LEU384, TYR415 MET414, PRO197, TYR448	CYS366, ARG200
**Nesbuvir**	−15.42	CYS316, SER368, ASP319, ARG200, LEU314	CYS366, SER365, LEU384, PHE193, CYS316, LEU204, VAL321, SER365, TYR448	MET414, CYS316

**Table 2:** ADMET, drug-likeness, and medicinal chemistry profiles of the lead compounds reported in this study.

ADMET and Drug-Likeness Profile	BF-9	BF-12	BF-13
LogS	−4.022	−5.624	−5.157
LogD	2.544	3.786	4.019
TPSA	85.420	72.950	72.950
HIA	+ive	+ive	+ive
AMES Toxic	No	Yes	Yes
MDCK cells permeability	Medium	Low	Low
Lipinski’s Rule	Accepted	Accepted	Accepted
Golden Triangle	Accepted	Accepted	Accepted
BBB penetration	+ive	+ive	+ive
Acute Toxicity Rule	0-Alerts	0-Alerts	0-Alerts

**Table 3:** MMPB/GBSA binding free energy calculation of the three ligand–protein complexes. All values are in Kcal/mol.

Energy Parameter	BF-9+NS5B Complex	BF-12+NS5B Complex	BF-13+NS5B Complex
**MM-GBSA**			
Van der Waals	−69.25	−70.06	−65.22
Electrostatic	−28.09	−29.10	−25.97
Delta G_gas_	−97.34	−99.16	−91.19
Delta G_solv_	20.01	21.00	19.88
Delta_Total_	−77.33	−78.16	−71.31
**MM-PBSA**			
Van der Waals	−69.25	−70.06	−65.22
Electrostatic	−28.09	−29.10	−25.97
Delta G_gas_	−97.34	−99.16	−91.19
Delta G_solv_	22.00	23.77	20.11
Delta_Total_	−75.34	−75.39	−71.08

**Table 4:** Free energy decomposition analysis to highlight the most contributing residues that stabilize the complexes.

Residues	Complex		
	BF-9	BF-12	BF-13
Arg200	−3.04	−4.20	−3.08
Arg386	−1.67	−1.08	−1.63
Asn369	−1.34	−1.05	−1.64
Asp319	−1.02	−1.11	−1.05
Cys316	−1.10	−1.64	−1.60
Cys316	−1.06	−1.00	−1.04
Cys366	−4.62	−3.10	−2.85
His467	−1.03	−1.05	−1.12
Leu384	−1.36	−1.14	−1.67
Met414	−3.2	−5.36	−3.36
Phe193	−1.36	−1.05	−1.67
Pro197	−1.10	−1.05	−1.87
Ser365	−1.05	−1.36	−1.45
Ser368	−1.05	−1.41	−1.36
Tyr415	−1.03	−1.01	−1.0
Tyr555	−2.68	−1.67	−1.39
Val370	−1.02	−1.09	−1.54

**Table 5:** The HOMO-LUMO and related energies of **BF-9, BF-12, and BF-13** (in eV).

Parameters	BF-9	BF-12	BF-13
E_total_	−37,010.927	−38,200.867	−37,639.591
E_HOMO_	−5.961	−6.008	−5.914
E_LUMO_	−1.913	−1.686	−1.865
ΔE	4.048	4.322	4.049
Ionization potential (IP = −E_HOMO_)	5.961	6.008	5.914
Electron affinity (A = −E_LUMO_)	1.913	1.686	1.865
Chemical potential (µ = −(I + A)/2)	−3.937	−3.847	−3.890
Hardness (η = (I − A)/2)	2.024	2.161	2.025
Mulliken electronegativity (χ = (I + A)/2) [60]	3.937	3.847	3.890
Softness (S = 1/2η)	0.247	0.231	0.249
Electrophilicity index (ω = µ^2^/2η) [61]	3.828	3.419	3.768
Maximum charge transfer (ΔN_max_ = (I + A)/2(I − A)) [62]	0.973	0.890	0.960

## Data Availability

All the data are contained in the manuscript and Supplementary Materials.

## Supplementary Materials

The following supporting information can be downloaded at https://www.mdpi.com/article/10.3390/biomedicines11113085/s1; Table S1: Binding Affinities Scores of Benzofuran-oxadiazole and Triazoles **BF1**–**BF15**.
