# Supplementary material for: An Exploration of the Inhibitory Mechanism of Rationally Screened Benzofuran-1,3,4-Oxadiazoles and-1,2,4-Triazoles as Inhibitors of NS5B RdRp Hepatitis C Virus through Pharmacoinformatic Approaches
Source: Biomedicines. 2023 Nov 17;11(11):3085. doi: 10.3390/biomedicines11113085 (PMC10669698; doi:10.3390/biomedicines11113085)
Supplement: Supplementary file 1 [file biomedicines-11-03085-s001.zip › biomedicines-2683412-supplementary.pdf]

An Exploration of the Inhibitory Mechanism of Rationally Screened Benzofuran-1,3,4-Oxadiazoles and-1,2,4-Triazoles as Inhibitors of NS5B RdRp Hepatitis C Virus through Pharmacoinformatic Approaches

Ali Irfan[1], Shah Faisal[2], Sajjad Ahmad[3,4,5], Muhammad Jawwad Saif* [6], Ameer Fawad Zahoor[1], Samreen Gul Khan[1], Jamila javid[7], Sami A. Al-Hussain[8], Muhammed Tilahun Muhammed[9], Magdi E. A. Zaki[8*]

Table S1. Binding Affinities Scores of Benzofuran-oxadiazole and Triazoles **BF1-BF15**

Compounds	Structures	Binding Energies with the PS-II allosteric site of HCV NS5B Polymerase
**BF-1**	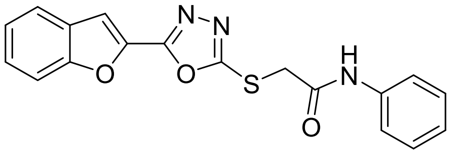	-13.35 Kcal/mol
**BF-2**	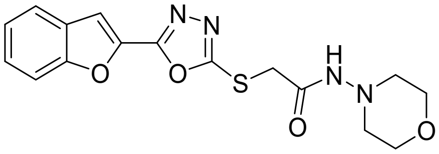	-12.87 Kcal/mol
**BF-3**	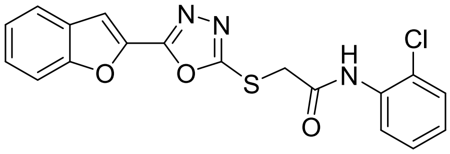	-13.45 Kcal/mol
**BF-4**	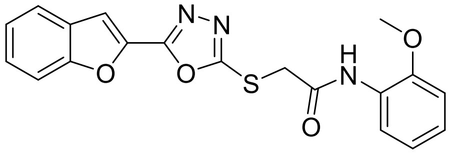	-12.63 Kcal/mol
**BF-5**	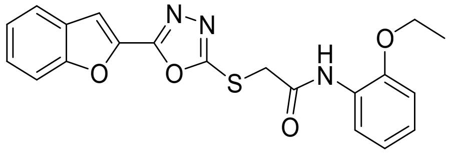	-14.04 Kcal/mol

**BF-6**	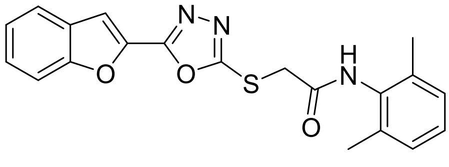	-13.23 Kcal/mol
**BF-7**	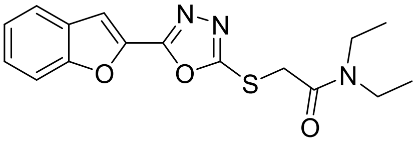	-13.97 Kcal/mol
**BF-8**	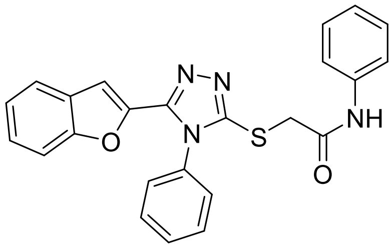	-14.19 Kcal/mol
**BF-9**	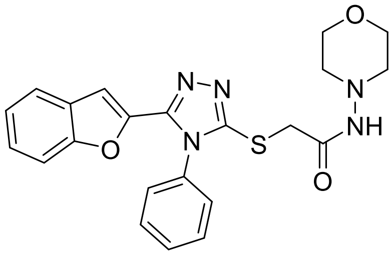	-16.09 Kcal/mol
**BF-10**	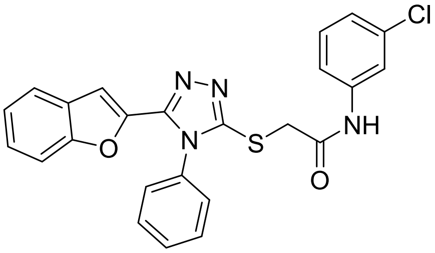	-14.93 Kcal/mol
**BF-11**	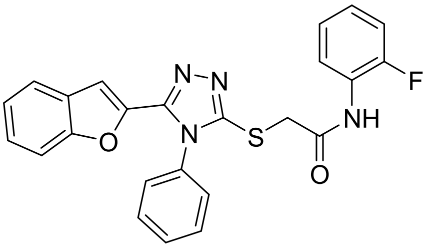	-14.88 Kcal/mol

**BF-12**	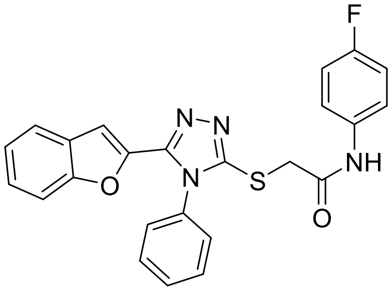	-15.75 Kcal/mol
**BF-13**	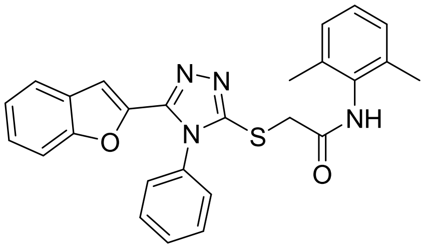	-15.82 Kcal/mol
**BF-14**	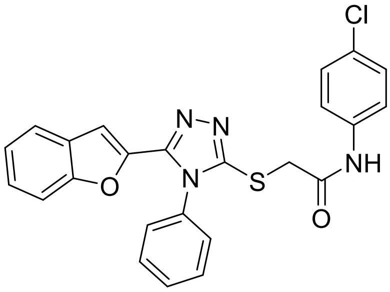	-14.11 Kcal/mol
**BF-15**	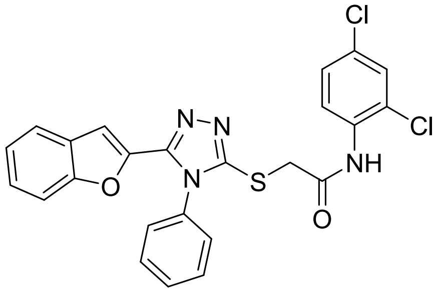	-14.84 Kcal/mol
**Benzofuran based standard reference NS5B inhibitors drug Nesbuvir**	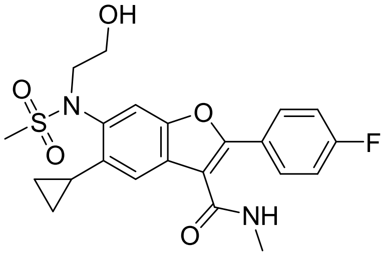	-15.42 Kcal/mol.
